# The Treatment of Infantile Paralysis

**Published:** 1902-04-12

**Authors:** 


### THE TREATMENT OF INFANTILE PARALYSIS.

Speaking lately at the Polyclinic on the subject of infantile paralysis, Dr. James Taylor referred to the treatment of this condition, dividing it into two parts, namely, that which is required at the onset of the paralysis and that which is to be employed later on, when all that can be done is to attempt to restore the function of the paralysed parts. As to the treatment at the time of the attack, this must be on general lines, as if one had to do with a general fever, the great point being not to overlook the possibility of any case of illness in a child, in which high temperature is associated with pain in the limbs, being one of commencing infantile paralysis. It often happens that it is only on a child beginning to get about again after a febrile attack that the paralysis which explains the nature of the preceding fever is discovered. There is no specific drug to be recommended at this stage, although salicylate of soda naturally occurs to mind as a means of neutralising the poison to which the condition may be due, or of relieving the pain that may be present. Later on, massage and passive movements come first in importance so as to prevent contractures and to favour the nutrition of the muscles. Then comes electricity; but, important as this is, it is far less important than massage. Its object is to produce a contraction in those muscles which no longer respond to voluntary efforts. It is, indeed, a mode of giving exercise to these muscles. The constant current is the form to employ, and it is very important to make the child well accustomed to the apparatus and the contact of the poles before any current is turned on, so as to avoid all fright and screaming. If one can produce a visible contraction of the paralysed muscles that is enough, but even though no visible contraction may occur, if one is sure that a good current is passing through the muscles one may be pretty confident that they are responding so far as they are capable of so doing, which is the great point. Thus, although the treatment falls into very small compass, it is important that it should be fully and carefully carried out.

